# Successful large caloric deficit with high protein modification diet and intensive aerobic and resistance training with progressive overload in adult patient with significant coronary artery disease: a case report

**DOI:** 10.3389/fcvm.2026.1737431

**Published:** 2026-02-04

**Authors:** Reynard Laysandro, Elbert Aldrin Harijanto, Nicky Alexandra Sie

**Affiliations:** 1 Department of Internal Medicine, Faculty of Medicine, Universitas Indonesia – Dr. Cipto Mangunkusumo General Hospital, Jakarta, Indonesia; 2 Department of Sport Medicine, Orthosports and Wellness Center Premier Bintaro Hospital, Tangerang, Indonesia; 3 Department of Cardiology and Vascular Medicine, Faculty of Medicine, Universitas Indonesia – National Cardiovascular Centre Harapan Kita, Jakarta, Indonesia

**Keywords:** caloric deficit high protein diet, cardiac rehabilitation, coronary artery disease, exercise, intensive lifestyle intervention, obesity

## Abstract

**Background:**

Lifestyle modification plays a central role in obesity and cardiometabolic disease management; however, its application in patients with obstructive coronary artery disease (CAD) is typically cautious due to safety concerns. Caloric restriction with a high protein diet and high-intensity exercise has not been well studied in this setting.

**Case presentation:**

A 43-year-old man with Class III obesity (BMI 43.8 kg/m²), uncontrolled hypertension and severe proximal LAD stenosis (CAD-RADS 4) presented with shortness of breath for evaluation. He declined percutaneous coronary intervention and chose structured intensive lifestyle therapy. Baseline data: waist 125 cm, BP 185/100 mmHg, visceral fat ∼40%, LDL 1.51 mmol/L, HDL 0.97 mmol/L, HbA1c 5.3%, stress METS 6.3 without ischemia.

**Management:**

Under weekly multidisciplinary supervision (internal medicine, cardiology, nutrition, sports medicine), he followed progressive caloric restriction with a high protein diet and high-intensity aerobic plus resistance exercise over 10 months. Usual cardiovascular medical therapy was continued. Monitoring included vitals, ECG, electrolytes, lipids, and exercise tolerance.

**Outcome:**

The patient lost 50 kg (41% of baseline) with BMI 25.8 kg/m², waist 85 cm, visceral fat 12%. Functional capacity improved (METS 6.30–11.5), HDL increased (0.97–1.63 mmol/L), HbA1c decreased (5.3%–4.9%), and blood pressure improved (185/100 to 140/85 mmHg). However, LDL and total cholesterol rose (LDL 1.51–3.44 mmol/L; total cholesterol 3.32–5.47 mmol/L). LDL rose consistent with fat mobilization physiology during diet and exercise. No arrhythmia or ischemic ECG changes were observed. The patient remained asymptomatic and entered maintenance training.

**Conclusion:**

Extreme supervised lifestyle intervention may be feasible in carefully selected high-risk CAD patients. Standard moderate programs remain recommended; extreme strategies require intensive medical oversight.

## Introduction

Obesity is a major global health burden and a key modifiable risk factor for cardiometabolic disease, including coronary artery disease (CAD). Excess adiposity exacerbates atherogenesis, endothelial dysfunction, systemic inflammation, and metabolic dysregulation, significantly increasing cardiovascular morbidity and mortality. Conventional management strategies comprising caloric restriction, pharmacotherapy, and structured exercise recommend gradual weight reduction of 5%–10% over six months, typically through moderate caloric deficit and aerobic exercise under clinical supervision (1–3).

Although intensive lifestyle modification programs have demonstrated efficacy in improving cardiometabolic profiles, most guidelines emphasize progressive rather than aggressive approaches, particularly in patients with established CAD (4–6). Extreme caloric restriction with high protein diet and high-intensity exercise have rarely been evaluated in individuals with high-risk coronary lesions due to concerns regarding arrhythmia, myocardial ischemia, hemodynamic instability, electrolyte imbalance, and adverse metabolic adaptations (7–9). Consequently, evidence on the safety and clinical response to highly intensive weight-loss strategies in patients with obstructive coronary disease remains limited.

Here we report a case of a 43-year-old male with severe obesity (BMI 43.8 kg/m²) and significant coronary artery stenosis who declined percutaneous coronary intervention (PCI) and instead underwent a supervised program combining extreme caloric deficit with high protein diet and high-intensity physical training. The case provides insight into physiological adaptation, metabolic changes, and cardiovascular safety considerations when implementing highly intensive lifestyle interventions in a high-risk CAD population.

## Case presentation

**Figure 1:**
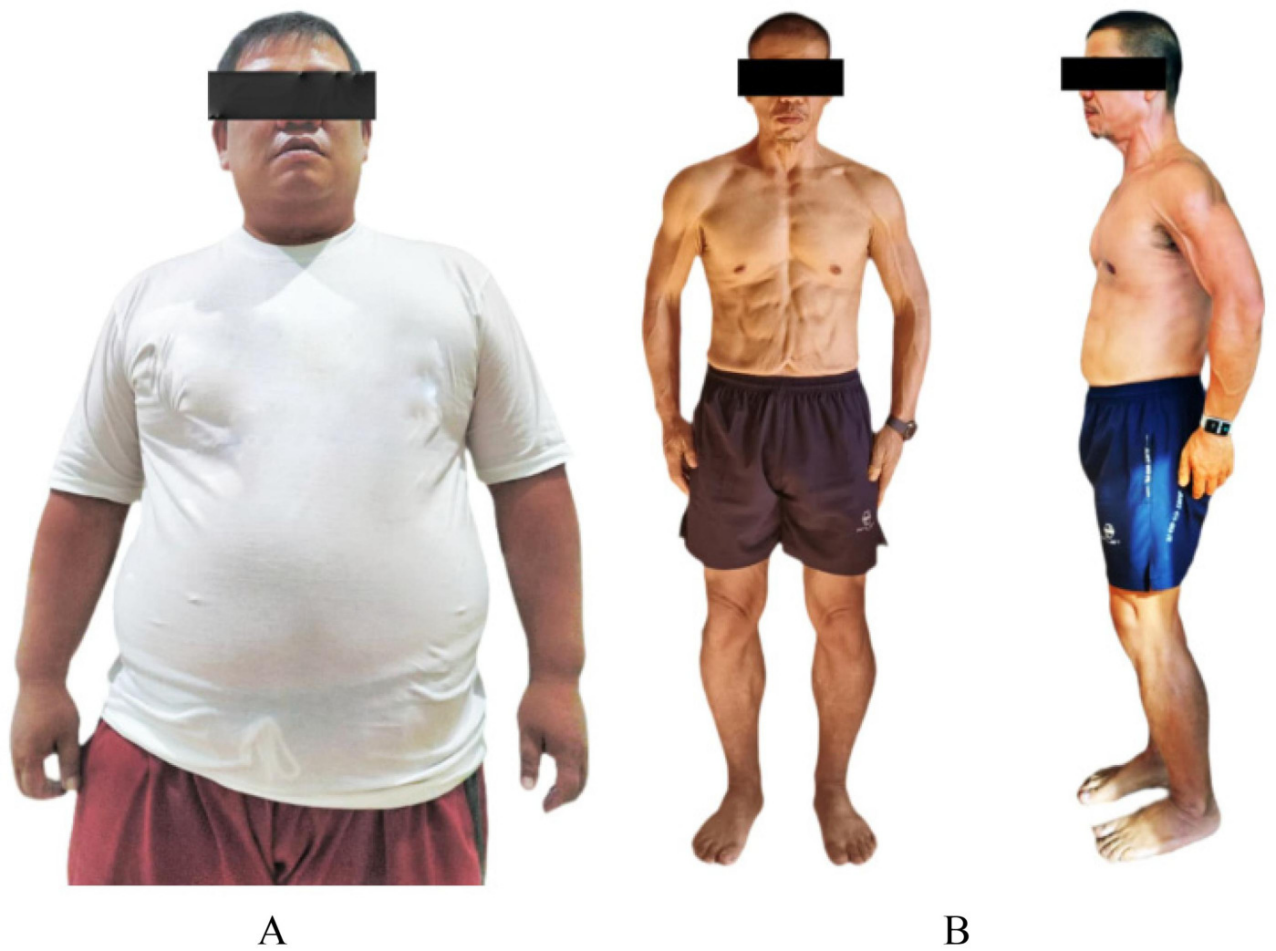
Body composition change from baseline to 10 months of supervised high-intensity lifestyle intervention in a patient with severe CAD. BMI at diagnose of 43.8 kg/m^2^ **(A)**, and BMI at 10 months of 25.81 kg/m^2^ **(B)**.

A 43-year-old Asian male underwent medical evaluation with shortness of breath during exercise. He denied dyspnea, palpitation and syncope. He reported lifelong obesity, sedentary lifestyle, and poorly controlled hypertension. He stated that his body condition had been similar since elementary school and denied any history of weight loss. The patient reported a long-standing struggle with weight since childhood and had attempted multiple conventional weight-loss programs in the past, including calorie-restricted diets and intermittent exercise, without sustained success. He expressed a strong personal motivation to improve his health following a family history of premature cardiovascular disease and his father's fatal myocardial infarction at age 55. The patient worked in an office-based administrative role, led a predominantly sedentary lifestyle, he denied smoking, alcohol and drug abuse. He had history of uncontrolled hypertension and take amlodipine occasionally. There was no history of diabetes, dyslipidemia diagnosis, or thyroid disease. On physical examination, the patient appeared in good condition, with a pyknic body habitus. Vital signs showed a blood pressure of 185/100 mmHg, heart rate of 98 beats per minute (strong and regular), respiratory rate of 21 breaths per minute, and body temperature of 36.8 °C. Anthropometric measurements were: height 167 cm, weight 122 kg, and waist circumference 125 cm. Body fat percentage was calculated using dual-energy x-ray absorptiometry (DXA) at 40% (Figure 1A).

Stress test showed METS 6.3, peak HR 155 bpm, BP 200/110 mmHg, no ischemia. Coronary CTA showed severe proximal LAD stenosis (70%–99%, CAD-RADS 4). Laboratories showed mild leukocytosis (11,000/µL), HDL 0.97 mmol/L, HbA1c 5.3%, normal renal & hepatic function. The patient declined PCI and consented to intensive monitored lifestyle therapy. Medications included amlodipine, rosuvastatin, clopidogrel, and allopurinol. Weekly clinical reviews were performed.

## Timeline

Table 1.

**Table 1:** Timeline of clinical course, intervention, and outcomes.

Time	Clinical status & evaluation	Intervention	Monitoring	Key findings/outcome
Baseline (Month 0)	CAD-RADS 4 (70%–99% proximal LAD stenosis), BMI 43.8 kg/m², waist 125 cm, visceral fat 40%, BP 185/100 mmHg, HR 98 bpm	Patient refused PCI; opted for supervised lifestyle intervention	CTA, ETT, Labs	Stable, no acute ischemic symptoms; high cardiovascular risk, no injury/ adverse event
Week 1–4	Physical tolerance screening; cardiovascular risk stratification	Initiation Phase: large deficit calorie diet (1,500 kcal/day), aerobic and resistance training, observed by physician	Vital Sign, ECG during exercise	Tolerated training; no adverse cardiovascular symptoms, no injury/ adverse event
Month 3	Weight decreased 10 kg; waist decrease 15 cm; BP improved;	Progressively increased intensity, continued LCD & supervised training	Vital Sign, ECG during exercise	Improved stamina; no arrhythmias or ischemic signs, no injury/ adverse event
Month 6	Weight decreased 30 kg; visceral fat decreased markedly	Transition Phase: 2,000 kcal/day; high-protein, low-carbohydrate diet (10% carb, 50% protein, 40% fat), intensive exercise 6×/week	Vital Sign, ECG during exercise	No complications; significant metabolic improvement, no injury/ adverse event
Month 8–9	Cardiorespiratory performance increased; BP and HR further improved	Continuation of intensive training & structured nutrition, supplementation (K2, D3, omega-3, niacin)	Vital Sign, ECG during exercise	No musculoskeletal or cardiovascular adverse events, no injury/ adverse event
Month 10	Weight decreased 50 kg (41% loss); BMI 25.8 kg/m²; visceral fat 12%; waist 85 cm	Maintenance plan initiated	ETT, Labs	Stable labs; no electrolyte abnormalities; preserved cardiac function, no injury/ adverse event
Month 12 (Follow-up)	HDL increased, HbA1c decreased, LDL/total cholesterol increased (lipid redistribution), METS 13.5, max HR 164 bpm, BP 140/85 mmHg	Maintain combined aerobic and resistance training; adjusted calories 2,000–2,500 kcal/day	Tele-follow up	Excellent functional recovery; normal ECG; strong exercise capacity; asymptomatic, no injury/ adverse event

CTA, CT Angiography; ETT, Exercise Tolerance Test; ECG, Electrocardiography.

### Investigations

Coronary angiography performed as part of the check-up revealed calcified plaques at the proximal and mid-LAD (Left Anterior Descending) junction, causing severe stenosis (70%–99%) (CAD-RADS 4) (Table 2). Laboratory tests showed leukocytosis (11,000/µL), low HDL cholesterol (0.97 mmol/L) (Table 3), and decreased cardiac functional capacity (20%–30%) on exercise stress testing, with a maximum heart rate response of 155 bpm and a peak blood pressure of 200/110 mmHg. Oxygen consumption during activity appeared adequate, with a Metabolic Equivalent of Task (METS) score of 6.30. Electrocardiography showed no significant abnormalities (Table 4). Chest x-ray examination didn't show any significant abnormality. The patient was categorized with a high-risk cardiovascular event by SCORE2-OP scoring system with class III obesity, grade II hypertension

**Table 2:** CT angiography interpretation.

Variable	Description
Action steps	I.V. Omnipaque 350 50 mL given followed by 50 mL of saline. ECG-gated reconstruction performed. GTN puffs given prior to examination.
Coronary Artery Calcium Scoring	Total coronary artery calcium score is 360. Multiple focal calcified plaques are noted in the right and left coronary arteries and their branches. The probability of significant non-obstructive coronary artery disease is highly likely although obstructive disease is possible. The implication for cardiovascular risk is moderately high.
Result	Normal configuration of the coronary arteries with right coronary dominance noted. A calcified plaque at the proximal right coronary artery is causing moderate stenosis (50%–69%). Scattered calcified and soft plaques in the rest of the RCA are causing minimal to mild narrowing. A small calcified plaque at the left main coronary artery is causing minimal stenosis (1%–24%). A dense calcified plaque at the junction of the proximal and mid-LAD is causing severe stenosis (70%–99%), with calcified and soft plaques at the mid-LAD, causing moderate stenosis (50%–69%). Calcified and soft plaques at the distal LAD are causing minimal to mild narrowing. Calcified and soft plaques at the D1 branch are causing moderate stenosis (50%–69%). A dense calcified plaque at the circumflex artery are causing mild stenosis (25%–49%). Calcified and soft plaques at the circumflex artery are also noted. No focal lung lesions seen in the regions scanned. No pleural effusion.
Impression	Dense calcified plaque at the junction of proximal and mid LAD causing severe stenosis (70%–99%). In keeping with a CAD-RADS 4 lesion. Calcified and soft plaques at the mid-LAD causing moderate stenosis (50%–69%) – CAD-RADS 3. Calcified plaque at the D1 branch causing moderate stenosis (50%–69%) – CAD-RADS Calcified plaque at the proximal right coronary artery (RCA) causing moderate stenosis (50%–69%) – CAD-RADS 3. Calcified and soft plaques at the circumflex artery causing mild stenosis.

**Table 3:** Results of complete blood count, lipid and glucose profile, urinalysis, and electrolytes.

Variable	Pre treatment (Diagnose)	Post treatment (10 months)	Reference value	Units
WBC	11.0	6.4	4.0–11.0	×10^9^/L
Differential Count				
Neutrophil	64	55	54–62	%
Lymphocytes	23	35	20–40	%
Monocytes	7	6	4–10	%
Eosinophils	6	4	1–6	%
Basophil	0.5	0.5	0.0–1.0	%
RBC	5.6	4.8	4.4–5.9	×10^12^/L
Hemoglobin	15.9	13.8	14–18	g/dL
PCV	47	42	41–53	%
MCV	84	88	80–100	fL
MCH	28	29	27–34	pg
MCHC	34	33	31–36	g/dL
RDW-SD	40.9	43	37–46	fL
RDW-CV	13.5	13.2	11–16	%
Platelet Count	277	248	150–400	×10^9^/L
Electrolyte				
Sodium	140	138	136–145	mmol/L
Potassium	4.8	4.9	3.5–5.1	mmol/L
Chlorides	101.0	102.4	98.0–107.0	mmol/L
Carbon dioxide	27.4	25.9	22–29	mmol/L
Anion Gap	16.4	14.6	8.0–16.0	mmol/L
Uric Acid	333	351	202–417	nmol/L
Calcium	2.50	2.51	2.15–2.50	mmol/L
Phosphorus	1.11	1.24	0.81–1.45	mmol/L
Magnesium	0.88	0.75	0.66–1.07	mmol/L
Urea	5.56	9.51	2.76–8.07	mmol/L
Creatinine	91	97	59–104	mmol/L
eGFR	89	82	>60	ml/min/1.73 m^2^
Glucose	5.31	5.43	3.89–5.83	mmol/L
HbA1c	5.3	4.9	<5.7	%
Lipid Profile				
Cholesterol	3.32	5.47	<5.20	mmol/L
Triglycerides	1.85	0.89	<1.70	mmol/L
HDL Cholesterol	0.97	1.63	>1.45	mmol/L
Non-HDL Cholesterol	2.35	3.84	<3.40	mmol/L
LDL Cholesterol	1.51	3.44	<2.6	mmol/L
Total CHOL/HDL	3.42	3.36	<5.00	ratio
Urinalysis				
Color	Yellow	Light Yellow	Gradation	–
Appearance	Clear	Clear	Clear	–
Blood	Negative	Negative	Negative	–
Bilirubin	Negative	Negative	Negative	–
Urobilinogen	Normal	Normal	Normal	–
Ketone	Negative	Negative	Negative	–
Glucose	Negative	Negative	Negative	–
Protein	Negative	Negative	Negative	–
Nitrite	Negative	Negative	Negative	–
Leucocytes	Negative	Negative	Negative	–
pH	6.0	5.5	4.5–8.0	–
Specific Gravity	1.014	1.005	1.005–1.030	–
Microscopy				
WBC	0	0	0	/HPF
RBC	0	0	0	/HPF
Epithelial Cell	0	0	0–15	/HPF
Renal Tubular Cell	0	0	0–1	/HPF
Bacteria	Negative	Negative	Negative	–
Pathological Cast	0	0	0–1	/LPF

HPF, High Power Field; LPF, Low Power Field.

Reference intervals used in this report are identical to those reported in the supplementary laboratory table and follow laboratory-validated intervals consistent with international standards and major guideline bodies (10–14).

**Table 4:** Exercise stress test.

Phase	Stage	Time in stage	Speed (km/h)	Grade (%)	HR (bpm)	BP (mmHg)
Pre Treatment (Diagnose)						
Pretest	Supine	00:01				
	Standing	05:06	0.00	0.00	90	140/90
	Warm Up	00:14	1.60	0.00	87	140/90
Exercise	Stage I	03:00	2.70	10.00	118	160/100
	Stage II	03:00	4.00	12.00	142	180/100
	Stage III	00:39	5.40	14.00	155	180/100
Recovery		04:16	0.00	0.00	116	180/100
The patient exercised according to the BRUCE for 6:39 min:s, achieving a work level of Max. METS: 6.30. The resting heart rate of 86 bpm rose to a maximal heart rate of 155 bpm. This value represents 87% of the maximal, age-predicted heart rate. The resting blood pressure of 140/90 mmHg, rose to a maximum blood pressure of 200/110 mmHg. The exercise test was stopped due to Dyspnea, Exaggerated BP increase. **Interpretation** Summary: Resting ECG: normal. (Negative Ischemic Response) Functional Capacity: moderately decreased (20% to 30%). HR Response to Exercise: appropriate. BP Response to Exercise: resting hypertension—exaggerated response. Chest Pain: none. Arrhythmias: none. Fitness classification FAIR, functional class N-1						
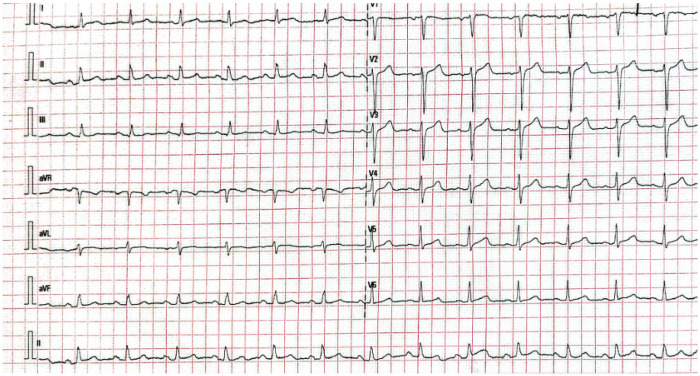						
Post Treatment (10 months)						
Pretest	Supine	01:50	1.60	0.00	68	130/80
Exercise	Stage I	03:00	2.70	10.00	98	130/90
	Stage II	03:00	4.00	12.00	113	140/90
	Stage III	03:00	5.50	14.00	146	150/90
	Stage IV	02:31	6.70	16.00	164	150/90
Recovery		04:17	0.00	0.00	101	160/90
The patient exercised according to the BRUCE for 11:30 min:s, achieving a work level of Max. METS: 11.5 The resting heart rate of 65 bpm rose to a maximal heart rate of 164 bpm. This value represents 92% of the maximal, age-predicted heart rate. The resting blood pressure of 130/80 mmHg, rose to a maximum blood pressure of 190/100 mmHg. The exercise test was stopped due to Target heart rate achieved, Dyspnea. **Interpretation** Summary: Resting ECG: normal. (Negative Ischemic Response) Functional Capacity: normal. HR Response to Exercise: appropriate. BP Response to Exercise: normal resting BP—exaggerated response. Chest Pain: none. Arrhythmias: none. Fitness classification GOOD, functional class N-1						
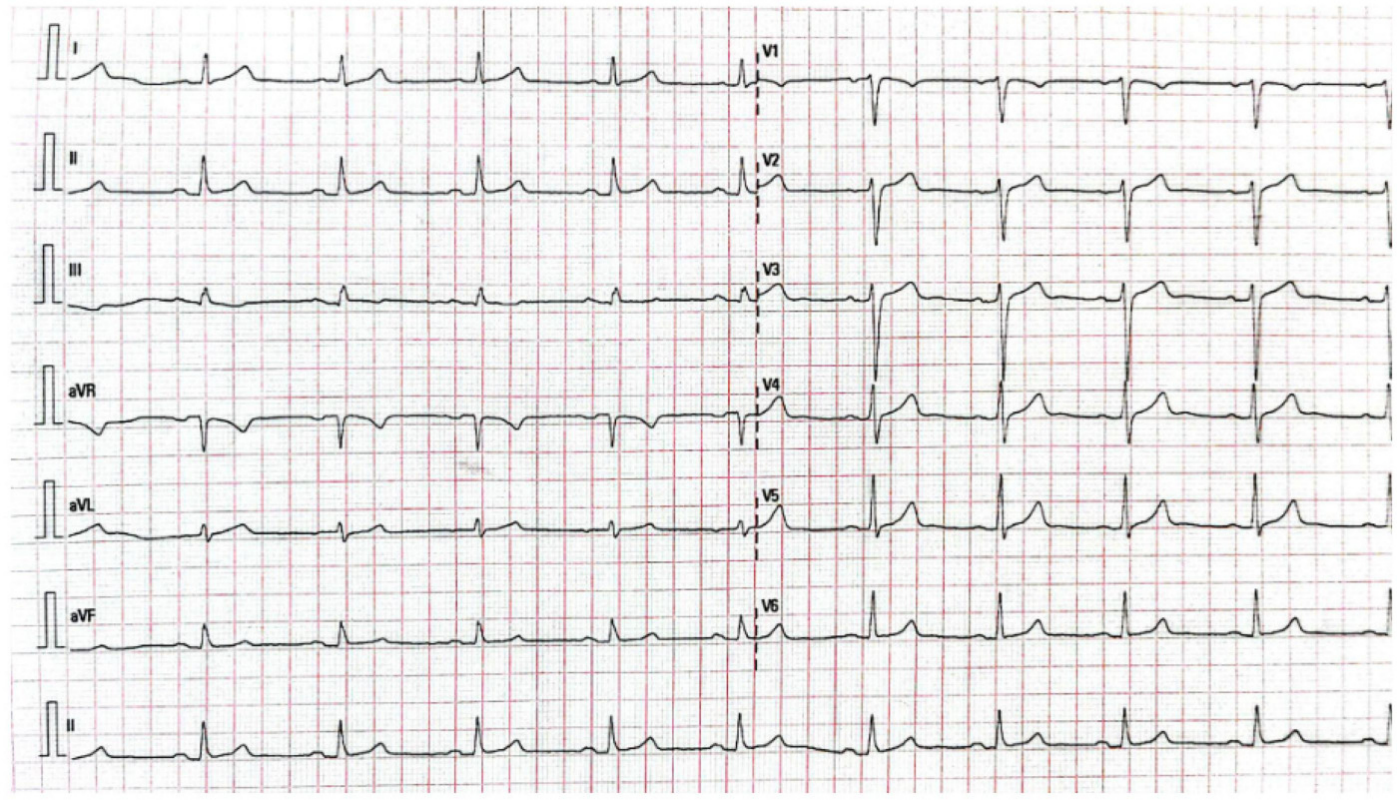						

### Management

The patient declined primary Percutaneous Coronary Intervention (PCI) and instead opted for a non-pharmacological management program (focus on lifestyle modification and concomitant drug intervention) by multidisciplinary team involving evaluation by an internist, cardiologist, nutritionist, and sports medicine specialist. The patient was prescribed preventive cardiovascular medications, including amlodipine 10 mg once daily, rosuvastatin 10 mg once daily and clopidogrel 75 mg once daily. The patient was also prescribed with allopurinol 100 mg irregularly to prevent hyperuricemia and physical exercise side effects (Table 5).

**Table 5:** Cardiovascular and adjunctive medications during the intervention period (17, 18, 24).

Medication	Class	Indication/rationale	Start time/phase	Initial dose	Dose changes	Adherence	Notes
Amlodipine	Dihydropyridine calcium channel blocker	Treatment of grade II hypertension and control of exercise BP response	Baseline (Month 0)	10 mg once daily	No dose change	High (>90% self-reported adherence)	Preferred agent due to resting hypertension and LV afterload reduction
Rosuvastatin	HMG-CoA reductase inhibitor	LDL-cholesterol reduction and secondary prevention in obstructive CAD	Baseline (Month 0)	10 mg once daily	Considered for up-titration to 20 mg after LDL rise at Month 6.	High	High-intensity statin therapy indicated in CAD; LDL rise prompted plan for dose escalation/combination therapy
Clopidogrel	P2Y12 inhibitor antiplatelet	Secondary prevention in obstructive CAD despite absence of PCI	Baseline (Month 0)	75 mg once daily	No change	High	Chosen due to CT-proven CAD-RADS 4 lesion; patient declined PCI; used as single antiplatelet therapy rather than DAPT
Allopurinol	Xanthine-oxidase inhibitor	Prevention of hyperuricemia associated with high-protein diet and intensive training	Phase 1 Month 1	100 mg intermittently	Dose not escalated	Intermittent supervised use	Also considered for antioxidant benefit (reduction in ROS); uric acid monitored regularly
Omega-3 supplement	Nutritional supplement	Triglyceride modulation and general cardiovascular risk modification	Phase 1	1,000 mg/day (EPA/DHA equivalent)	No change	Good	Not prescribed as lipid-lowering monotherapy; adjunct only
Vitamin D3	Supplement	Correction/prevention of deficiency during calorie restriction	Phase 1	1,000–2,000 IU/day	Adjusted based on serum level	Good	Not for CAD treatment; deficiency prevention
Vitamin K2	Supplement	Bone/vascular health support during rapid weight loss	Phase 2	90–180 mcg/day	No change	Good	Supportive therapy
Niacin	Supplement	HDL-C support and lipoprotein modification	Phase 2	500 mg/day	No change	Moderate	Used as adjunct; flushing discussed with patient
Antianginal therapy (not initiated)	Nitrates/β-blocker	Considered due to severe LAD stenosis or ACS	—	—	—	—	Not started because patient remained asymptomatic, normal ETT ischemia response, and BP controlled. To be initiated if angina developed

The exercise and nutrition program lasted 10 months and was divided into three phases: an initiation phase (6 months) and a transition phase (4 months), followed by a maintenance phase aimed at preserving the patient's physical condition (Table 6). The program was directly supervised by physicians and coaches with variable types of exercise (Table 7). The patient underwent routine evaluations during exercise, and assessments of physical and cardiovascular compliance were performed at the end of the second phase. During the program, the patient maintained a total sleep duration of 8 ± 1 h daily.

**Table 6:** Exercise and nutrition therapy.

Therapy	Phase 1 (Initiation)	Phase 2 (Transition)	Phase 3 (Maintenance)
Duration	6 months	4 months	>10 months
Exercise regimen	Cardio exercise 1–2 h, 6×/week. Each session lasting 2 h.	Combination of 40% cardio and 60% resistance training. Cardio lasting 1 h. Resistance training lasting 2 h.	Combination of 30% cardio and 70% resistance training. Cardio lasting 1 h. Resistance training lasting 2 h.
Nutritional regimen	Daily caloric restriction to 2 meals per day (1,500 kcal per day) Elimination of sugar, flour, and ultra-processed foods. Macronutrient distribution: 30% carbohydrate, 40% protein or 1.6–2.0 g/kg//day, 30% fat. Fiber intake 25–35 g/day.	Daily calories increased to 2 meals per day (2,000 kcal per day) Continued elimination of sugar, flour, and ultra-processed foods. Macronutrient distribution: 10% carbohydrate, 50% protein or 2.0–2.5 g/kg/day, 40% fat.	Daily caloric intake 2 meals per day (2,000–2,500 kcal per day) Continued elimination of sugar, flour, and ultra-processed foods. Macronutrient distribution: 30% carbohydrate, 40% protein or 1.6–2.0 g/kg//day, 30% fat. Fiber intake 25–35 g/day
Outcomes	Weight reduction of 30 kg with body fat 30% (24.6% reduction from baseline).	Additional 20 kg weight reduction with body fat 12% (total 41% reduction from baseline).	Body weight stabilized at 72 kg.

**Table 7:** Exercise regimen details.

Type of Exercise	Phase 1 (Initiation)	Phase 2 (Transition)	Phase 3 (Maintenance)
Chest			
Upper chest: Incline bench press, low-to-high cable fly	3 × 12 times (or tolerated limit), 6 kg load	3 × 12 (or tolerated limit), 12 kg load	3 × 12 (or tolerated limit), 15 kg load
Mid chest: Bench press, mid cable fly	3 × 12 (or tolerated limit), 8 kg load	3 × 12 (or tolerated limit), 20 kg load	3 × 12 (or tolerated limit), 27.5 kg load
Lower chest: Decline bench press, high-to-low cable press	3 × 12 (or tolerated limit), 10 kg load	3 × 12 (or tolerated limit), 30 kg load	3 × 12 (or tolerated limit), 40 kg load
Back			
Lats, rhomboid: Straight-arm lat pulldown	3 × 12 (or tolerated limit), 8 kg load	3 × 12 (or tolerated limit), 15 kg load	3 × 12 (or tolerated limit), 20 kg load
Lats, traps, rhomboid: Reverse-grip lat pulldown	3 × 12 (or tolerated limit), 8 kg load	3 × 12 (or tolerated limit), 15 kg load	3 × 12 (or tolerated limit), 20 kg load
Rhomboid, teres, supraspinatus: Face pull	3 × 12 (or tolerated limit), 8 kg load	3 × 12 (or tolerated limit), 15 kg load	3 × 12 (or tolerated limit), 20 kg load
Shoulder			
Trapezius: Shrug	3 × 12 (or tolerated limit), 4 kg load	3 × 12 (or tolerated limit), 8 kg load	3 × 12 (or tolerated limit), 10 kg load
Anterior deltoid: Front raises	3 × 12 (or tolerated limit), 4 kg load	3 × 12 (or tolerated limit), 8 kg load	3 × 12 (or tolerated limit), 10 kg load
Middle deltoid: Lateral raises	3 × 12 (or tolerated limit), 4 kg load	3 × 12 (or tolerated limit), 8 kg load	3 × 12 (or tolerated limit), 10 kg load
Posterior deltoid: Bent-over lateral raises	3 × 12 (or tolerated limit), 4 kg load	3 × 12 (or tolerated limit), 8 kg load	3 × 12 (or tolerated limit), 10 kg load
Arm			
Biceps (short head): Preacher curl	3 × 12 (or tolerated limit), 4 kg load	3 × 12 (or tolerated limit), 6 kg load	3 × 12 (or tolerated limit), 8 kg load
Biceps (long head): Biceps curl	3 × 12 (or tolerated limit), 4 kg load	3 × 12 (or tolerated limit), 6 kg load	3 × 12 (or tolerated limit), 8 kg load
Triceps: Rope push-down	3 × 12 (or tolerated limit), 4 kg load	3 × 12 (or tolerated limit), 6 kg load	3 × 12 (or tolerated limit), 8 kg load
Forearm: Wrist curl	3 × 12 (or tolerated limit), 4 kg load	3 × 12 (or tolerated limit), 6 kg load	3 × 12 (or tolerated limit), 8 kg load
Leg			
Quadriceps: Squat, leg extension (machine)	3 × 12 (or tolerated limit), 60 kg load	3 × 12 (or tolerated limit), 100 kg load	3 × 12 (or tolerated limit), 120 kg load
Hamstrings: Deadlift, hamstring curl/leg curl	3 × 12 (or tolerated limit), 20 kg load	3 × 12 (or tolerated limit), 40 kg load	3 × 12 (or tolerated limit), 60 kg load
Gastrocnemius, soleus: Standing or seated calf raises	3 × 12 (or tolerated limit), 20 kg load	3 × 12 (or tolerated limit), 40 kg load	3 × 12 (or tolerated limit), 60 kg load
Tibialis anterior: Tibialis Anterior Raise	3 × 12 (or tolerated limit), 20 kg load	3 × 12 (or tolerated limit), 40 kg load	3 × 12 (or tolerated limit), 60 kg load

Repetitions were performed to the patient’s tolerated limit. Load was progressively increased by 1–2 kg per week. Post-exercise evaluations were conducted to assess vital sign, cardiometabolic assessment and injury risk after each session.

Exercise prescription was described using the FITT-VP (Frequency, Intensity, Time, Type, Volume, Progression) framework. Training intensity was individualized based on results of the baseline treadmill exercise test and resting blood pressure control (Table 8) (15, 16).

**Table 8:** Aerobic and resistance training prescription structured using the FITT-VP framework (15, 16).

Component	Aerobic training	Resistance training
Frequency	5–6 days per week	2–3 non-consecutive days per week
Intensity	60%–80% heart rate reserve (HRR)	50%–70% of estimated 1-repetition maximum (1-RM)
	Corresponding to 5–7 METs	
	Borg RPE 13–15	
Time (per session)	Phase 1: 30–60 min	Typically 20–40 min
	Phase 2 onward: 60–90 min	
Type	Treadmill walking and cycle ergometry	Major muscle group multi-joint exercises
High-Intensity Interval Component (when applied)	Intervals of 3–5 min at 80–90% HRR	—
	Alternated with 3–4 min at 50%–60% HRR	—
	Total interval duration ≤30 min/session	—
Volume	Initial weekly aerobic volume: 300–360 min	2–3 sets of 10–15 repetitions per exercise
	Progressed to 450–540 min/week	8–10 exercises targeting major muscle groups
Progression	5%–10% weekly increase in duration prior to intensity progression	Load increased when 15 repetitions performed without symptoms
Technique & Safety	BP monitored; interval progression only after tolerance	Slow controlled breathing; avoidance of Valsalva maneuver
Termination Criteria	RPE > 15, angina, dizziness, SBP ≥ 220 or DBP ≥ 110, arrhythmia, abnormal ECG	Pain, dizziness, abnormal hemodynamic response
Note	Training initiated after BP control	Training to failure was not used

FITT-VP, Frequency, Intensity, Time, Type, Volume, Progression.

All exercise sessions were supervised by a cardiologist and sports medicine specialist**.** Telemetry ECG monitoring was used during early sessions and during increases in intensity. 12-lead resting ECG was repeated weekly. Exercise ECG was reviewed for arrhythmias, ST-segment changes, and repolarization abnormalities. Blood pressure was measured at rest before exercise, at peak workload, every 5–10 min during prolonged sessions and during recovery. Exercise was terminated if systolic BP ≥ 220 mmHg or diastolic BP ≥ 110 mmHg, fall in systolic BP ≥ 10 mmHg with increasing workload, moderate angina, new limiting dyspnea, dizziness, presyncope, ≥2 mm horizontal/down sloping ST depression, complex ventricular arrhythmias. The patient was instructed to report chest pain using a standardized angina checklist. An automated external defibrillator and emergency kit were available onsite. Antihypertensive therapy was optimized before training progressed beyond 60% HRR. Medication regimens were reviewed weekly and adjusted by the treating cardiologist (15, 16).

As part of the regimen, the patient regularly consumed probiotics with a daily intake of 200 mL of kefir, one tablespoon of flaxseeds, and one tablespoon of chia seeds. Supplementation with vitamin K2, vitamin D3, omega-3, and niacin was provided to meet micronutrient and amino acid requirement (Table 5). In addition, the patient consumed three boiled eggs every morning and incorporated moringa leaves as an additional source of protein. Beyond caloric intake, careful attention was given to macronutrient and micronutrient composition to minimize potential adverse effects related to the weight reduction.

### Outcome and follow-up

Throughout the program, the patient remained hemodynamically stable with no episodes of syncope, new angina, arrhythmia, electrolyte imbalance, or rhabdomyolysis. During early training sessions, continuous ECG telemetry was used with HR targets of 60%–80% HRR and reached 80%–90% HRR. Borg RPE 13–15. The patient had good exercise tolerance on ETT without ischemic ST depression, controlled resting blood pressure and no complex arrhythmias.

After approximately 10 months of the program, the patient returned for clinical evaluation. He reported no significant complaints. On examination, the patient appeared well and displayed an athletic body habitus. Vital signs showed a blood pressure of 140/85 mmHg, heart rate of 64 bpm (regular and strong), respiratory rate of 18 breaths per minute, and body temperature of 36.9 °C. Physical examination was within normal limits, with a body weight of 72 kg (BMI 25.81 kg/m²) and a visceral fat level of 12% and a waist circumference of 85 cm. The patient had a weight reduction of 50 kg (Figure 1B).

Laboratory findings demonstrated a reduction in leukocyte count and an increase in HDL cholesterol (reaching the target normal range), as well as a decrease in HbA1c. However, total cholesterol and LDL cholesterol levels increased beyond the normal range (Table 3).

On exercise stress testing, the patient achieved stage 4, with improved oxygen consumption during exercise (METS 11.5). Cardiac capacity was within normal limits, with a maximum heart rate response of 164 bpm and peak blood pressure of 190/100 mmHg. There was no sign of presyncope and new angina. Electrocardiographic examination revealed no abnormalities (Table 4).

## Discussion

This case illustrates that a patient at high risk for severe coronary syndrome with comorbid obesity was able to physiologically compensate for an intensive dietary program and high-intensity exercise regimen. Serial clinical evaluations demonstrated favorable adaptation and positive outcomes throughout the intervention. Although current guidelines generally recommend initiating lifestyle interventions gradually and starting at low intensity (1), the program in this case was accelerated according to the patient's tolerance and functional capacity.

To date, there remains limited high-quality evidence supporting the safety of extreme caloric restriction combined with high-intensity training in adults with elevated cardiovascular risk. Such ultra-intensive lifestyle interventions (large caloric deficits and aerobic and resistance training) have not been extensively evaluated in symptomatic coronary artery disease populations, and therefore long-term safety cannot be presumed. Potential adverse effects include increased risk of electrophysiologic disturbances and arrhythmias [rapid weight loss can alter cardiac repolarization (QT and T-wave changes), heightening arrhythmia susceptibility (Level IIb, Grade B)] (2), as well as fluid and electrolyte imbalance, and myocardial stress [rapid weight fluctuation exceeding 10%–15% over a short period has been associated with catabolic stress, electrolyte shifts, and increased cardiovascular morbidity and mortality (Level IIb, Grade B)] (3).

Current cardiovascular prevention guidelines endorse diet and exercise as first-line therapy; however, they emphasize moderate targets (e.g., a caloric deficit of 500–750 kcal/day and ≥150 min of moderate-intensity aerobic activity weekly) aimed at a 5%–10% reduction in body weight (4, 5). Accordingly, strict supervision by a multidisciplinary team (internist, cardiologist, nutritionist, and sports medicine specialist) is essential in such intensive approaches. Several reports suggest that aggressive weight reduction can be performed safely, provided that close monitoring is implemented (electrolytes, ECG, renal function, and cardiovascular biomarkers) (2). Extreme diet and exercise protocols may be feasible in carefully selected patients under highly controlled settings; however, they should not be considered universally safe for high-risk populations. For this group, guideline-directed moderate-intensity intervention remains preferable, with gradual escalation to high intensity based on clinical tolerance and structured monitoring (2, 4, 5) (Table 9).

**Table 9:** Comparison between the patient’s modified nutritional therapy and guideline-based recommendations (17, 18, 20).

Aspect	Modified diet	Guideline	Notes
Weight-Loss Target	Very aggressive: 50 kg loss (41% of initial body weight) within 10 months.	5%–10% reduction of initial body weight within 6 months (AHA/ACC, ESPEN) to improve comorbidities, or >15% for class III obesity.	Weight loss exceeded the maximum guideline target; goal achieved with normalization of BMI and controlled blood pressure.
Caloric Intake	Phase 1: 1,500 kcal/day.	For class III obesity: 500 kcal/day deficit or consider low-calorie diet (LCD) 1,000–1,500 kcal/day under medical supervision.	Total calories aligned with guideline principles, yet caloric deficit applied aggressively to achieve target weight.
Macronutrient Composition	Phase 1: 30% carbs, 40% protein, 30% fat. 25–35 g fiber; Phase 2: 10% carbs, 50% protein, 40% fat. Phase 3: 30% carbs, 40% protein, 30% fat. 25–35 g fiber;	Balanced diet: 45%–50% carbs, 15%–20% protein, 30%–35% fat.	Deviates from standard ratios; extremely high protein and very low carbohydrate intake (especially Phase 2). This high-protein diet aimed to preserve lean body mass during extreme caloric deficit, with clinical supervision in specific settings.
Protein Intake	Very high: Phase 2 (50% of total daily energy), Phase 1 (40% of total daily energy).	1.2–1.5 g/kg ideal body weight/day (ESPEN); typically 15–25% of daily energy.	Protein intake intentionally increased to preserve lean body mass given extreme caloric deficit; exceeds guideline upper limit.
Food Quality & Supplementation	Elimination of sugar, flour, ultra-processed foods; daily consumption of kefir, flax seeds, chia seeds; supplementation (probiotic, antioxidants, vitamin K2, D3, omega-3, niacin).	Whole foods-based diet (fruit, vegetables, whole grains), low sugar/salt/saturated fat (AHA/ACC; Mediterranean/DASH). Most guidelines do not recommend non-prescription supplements (fish oil, vitamins) to reduce cardiovascular risk in CAD patients unless deficiency exists.	Aligned with the whole-food principle, but the supplementation strategy deviated from guideline-conservative approach; intended to mitigate extreme dietary risk in CAD.
Protein/Purine Metabolism	Very high protein (50% Phase 2); 3 boiled eggs/day; intermittent allopurinol 100 mg.	High-protein diets increase urea and uric acid, which may burden kidneys.	Urea increased (5.56–9.51 mmol/L); uric acid slightly increased (333–351 nmol/L) were high protein and extreme calorie deficit diet responses but within normal range; use of allopurinol demonstrated caution in hyperuricemia risk.
Systemic Inflammation (WBC/NLR)	Extreme weight loss (50 kg), intense exercise, probiotics/antioxidants consumption.	Class III obesity associated with chronic systemic inflammation (elevated WBC/NLR); weight loss reduces inflammation.	WBC and NLR reduction indicate improved inflammatory status due to obesity and cardiovascular risk modification.
Dyslipidemia Management (Cholesterol/LDL/HDL)	High protein/healthy fats (eggs, omega-3, chia/flax seeds); rosuvastatin 10 mg.	Statins to reduce LDL, especially in CAD patients. High cholesterol consumption can increase LDL in the hyper-responder population.	LDL increased despite statin therapy consistent with dietary hyper-responder phenotype. Although HDL improved, protein/fat sources and statin dose adjustment were required.
Endogenous Cholesterol Elevation	High-intensity training 6×/week, extreme caloric deficit, very low carbs (10%).	Intense exercise and caloric deficit turn into hormonal and metabolic responses such as increased cortisol and ketogenesis, fat mobilization from adipose tissue.	Physiologic response, cortisol and rise in acetyl-CoA (cholesterol precursor) from accelerated and huge fat mobilization promotes hepatic cholesterol synthesis and transport, contributing to increasing LDL.
Cholesterol Sensitivity	3 boiled eggs/day as protein source (cholesterol source).	Most individuals not cholesterol-sensitive; genetic hyper-responders show marked LDL rise with cholesterol intake.	Predisposition factor: marked LDL elevation despite statin use suggests hyper-responder phenotype; heightened sensitivity to dietary cholesterol contributed to LDL elevation.

In this case, physiologic parameters and weight reduction were closely monitored throughout the intervention. Extreme weight loss induces several metabolic and systemic adaptations, including reductions in basal metabolic rate (metabolic adaptation), hormonal shifts [characterized by decreased leptin, insulin, and thyroid hormone (T3) levels, alongside increases in ghrelin and cortisol which promote appetite and energy conservation], and hemodynamic changes such as reduced blood volume and lower ventricular filling pressures. These alterations may influence cardiovascular physiology by reducing cardiac workload, decreasing peripheral vascular resistance, and improving endothelial function, ultimately contributing to enhanced cardiorespiratory capacity. Additionally, substantial weight loss triggers adaptive thermogenesis as a compensatory mechanism to maintain energy balance (6, 7). Weight reduction exceeding 10% has been consistently associated with significant improvements in cardiovascular risk factors, particularly serum lipid profiles and blood pressure, across multiple observational studies (8).

Based on the patient's laboratory findings, there was an increase in HDL cholesterol and a reduction in HbA1c. However, elevations in LDL cholesterol and total cholesterol were also observed. Extreme weight loss can trigger substantial mobilization of adipose tissue fat stores, increasing the flux of free fatty acids to the liver and stimulating lipoprotein production, which may result in a transient rise in LDL or total cholesterol (lipid rebound effect). Rapid fat loss mobilizes large triglyceride reserves from adipose tissue, thereby increasing hepatic delivery of free fatty acids and subsequently promoting VLDL production and its conversion to LDL. Following a period of severe caloric restriction, caloric reintroduction may amplify hepatic lipogenesis and impair lipoprotein clearance, contributing to elevations in LDL and total cholesterol. Hormonal changes involving adipokines (e.g., leptin, adiponectin) and thyroid hormones further influence hepatic lipoprotein synthesis, while residual low-grade inflammation may modify lipid metabolism (6). Although LDL increased during the period of rapid weight loss, this finding should be interpreted cautiously. Potential contributors include diet composition, negative energy balance, altered hepatic cholesterol flux, and inter-individual variation in response to statin therapy. However, there were absence of ApoB and LDL particle data, making remain speculative and requires cautious interpretation. Further mechanistic work is required to clarify whether such LDL elevations reflect unfavorable atherogenic changes or benign transitional physiology during major weight reduction. In the context of obstructive CAD, this rise is clinically relevant and reinforces the need for close lipid monitoring and optimization of lipid-lowering therapy rather than being considered a benign or expected phenomenon (17, 18, 20, 21).

High-intensity exercise can enhance lipolysis and fat mobilization; however, in the setting of increased caloric intake or metabolic adaptive transition, compensatory lipogenesis may occur. Without precise synchronization of energy deficit and metabolic demands, a “lipid metabolism overshoot” may develop. Additionally, elevated intake of saturated fats or cholesterol, or substantial shifts in macronutrient composition (e.g., very low-carbohydrate diets), may increase LDL levels. Extreme carbohydrate restriction and macronutrient alterations have been shown to modify lipoprotein patterns, including increases in LDL-cholesterol and LDL particle size (Level Ia, Grade A recommendations) (9, 17).

Intensive lifestyle modification programs incorporating structured exercise can induce favorable adaptations in endothelial function, autonomic balance, inflammatory tone, and metabolic efficiency in patients with coronary artery disease. Vascular endothelial growth factor (VEGF) and stromal cell-derived factor-1*α* (SDF-1*α*) act synergistically to enhance endothelial progenitor cell (EPC) proliferation, migration, and differentiation while reducing apoptosis, whereas these effects are not extended to vascular smooth muscle cells. The findings highlight the interplay between angiogenic and chemokine signaling in vascular repair processes and suggest potential therapeutic strategies to support revascularization and inhibit vascular stenosis during exercise. Emerging pharmacologic and physiologic research demonstrates that structured exercise acts as a potent disease-modifying stimulus, interacting with signaling pathways related to vascular tone, lipid metabolism, mitochondrial function, and oxidative stress (22, 23). Our findings are consistent with this broader body of work, illustrating concordant improvements in blood pressure, functional capacity, and anthropometric parameters despite severe proximal coronary stenosis.

The use of cardiovascular medications in this patient, including antihypertensives, statins, and antiplatelet therapy (aspirin/clopidogrel), aligns with primary and secondary prevention guidelines, integrating pharmacotherapy with lifestyle modification. Pharmacologic management was tailored according to the patient's cardiovascular risk classification, consistent with the 2025 ESC recommendations. The prescription of antihypertensives, high-intensity statin therapy, and antiplatelet agents was appropriate for a high-risk profile, in accordance with Class Ia, Grade A recommendations (18).

The patient also received allopurinol therapy. Beyond its urate-lowering effect, allopurinol reduces production of reactive oxygen species (ROS), such as superoxide and hydrogen peroxide, which may confer benefit in mitigating oxidative stress associated with intensive physical training. However, allopurinol administration in this case was intermittent and closely supervised by a clinician, consistent with Class Ib, Grade A recommendations (19).

Recent guidelines additionally emphasize that pharmacologic therapy for obesity (such as GLP-1 receptor agonists) may be considered earlier in the treatment course, particularly in individuals at high cardiovascular risk, as an adjunct to lifestyle-based interventions (Class Ib, Grade A recommendations) (20).

The nutritional strategy in this case represented a modified approach compared with existing guidelines. While current obesity management guidelines typically recommend a 5%–10% reduction in body weight over six months (24), this patient achieved a 24.6% weight reduction, accompanied by favorable cardiovascular adaptation. Extreme macronutrient distribution (e.g., 10% carbohydrates and 50% protein) combined with high-intensity, high-volume exercise has not been extensively evaluated in high-risk cardiovascular populations. Notably, despite being classified as CAD-RADS 4 and falling into a high-risk category according to the 2025 ESC guidelines, the patient demonstrated excellent tolerance to both intensive exercise and a highly restrictive diet (18).

This favorable response was likely facilitated by close clinical supervision and targeted supplementation (e.g., vitamin K2, omega-3 fatty acids), administered with careful consideration of dosing, interactions, and evidence-based benefit (Class Ia, Grade A recommendations). However, such comprehensive multimodal therapy is not universally required for all patients and should be reserved for carefully selected individuals (25, 26). Although vitamin D supplementation has not consistently demonstrated cardiovascular benefit in patients with established coronary artery disease (Class Ia, Grade A recommendations) (27, 28), it was used in this case to maintain adequate baseline vitamin D status.

From a caloric standpoint, the patient's intake during Phase 1 (initiation; 1,500 kcal/day) and Phase 2 (transition; 2,000 kcal/day), falls within the range of a high deficit calorie diet or even maintenance intake in certain contexts (24, 29). This regimen was subsequently followed by Phase 3 (maintenance; 2,000–2,500 kcal/day). The relatively high caloric intake in later phases (despite substantial weight loss) reflects the patient's markedly elevated total daily energy expenditure (TDEE), driven by a large body mass and sustained high-intensity physical activity (six sessions per week). As such, a caloric deficit was still achieved (30).

According to current guidelines, a balanced dietary pattern typically consists of approximately 45%–50% carbohydrates, 15%–20% protein, and the remainder from fats (29). In this case, however, the macronutrient composition represented a modified high-protein, low-carbohydrate strategy, incorporating elements similar to the Dukan diet (31). This was most apparent during Phase 2, in which the dietary pattern became highly restrictive, with carbohydrate intake reduced to 10% and protein increased to 50% to preserve lean body mass and enhance satiety (24). Intermittent fasting play a roll in effective dietary intervention for slowing cardiometabolic aging process. Its related to the influences key for cardiometabolic risk factors like insulin sensitivity, inflammation and lipid metabolism (32).

Furthermore, the elimination of processed foods, sugars, and flour paralleled core principles of Mediterranean (DASH) dietary frameworks, emphasizing whole foods and supporting cardiovascular risk reduction (24). However, the markedly elevated protein proportion (50%) during Phase 2 may elicit metabolic responses such as increases in urea and uric acid levels, which represent physiological consequences of elevated protein turnover and treating with allopurinol medications (Table 7) (19, 29).

Patients undergoing this intensive lifestyle modification program must have preserved organ function (cardiac, renal, and hepatic), absence of decompensated comorbidities or arrhythmias, high adherence, close clinical supervision, meticulous monitoring of electrolytes and cardiovascular status. Ideal candidates include individuals in early middle adulthood, without unstable cardiovascular disease or severe metabolic derangements (e.g., renal failure, fluid overload), with adequate psychosocial support, and involvement of a multidisciplinary care team. Patients at high risk of complications (e.g., advanced cardiac disease, ventricular dysfunction) should be excluded or monitored with extreme vigilance. In obesity research, intensive interventions demonstrate greater effectiveness among individuals with severe obesity (BMI >35–40 kg/m^2^), substantial metabolic risk, and who are not elderly or burdened by advanced comorbidities (33).

In this case, the patient demonstrated improved cardiovascular performance during the stress test. Enhanced exercise tolerance (reflected by improved vital sign responses, reduced dyspnea during exertion, and higher METS) indicated robust cardiovascular compensation following the intervention program. These improvements can be attributed to reduced cardiac workload secondary to weight loss, enhanced endothelial and vascular function, decreased peripheral resistance, improved skeletal muscle oxidative capacity (including mitochondrial efficiency), and reductions in oxidative stress and inflammation, all of which facilitate greater VO₂ and METS achievement. Lower blood pressure and coronary arterial resistance improved myocardial perfusion during exertion. Both aerobic and resistance training contributed to superior cardiorespiratory reserve and cardiac–muscle efficiency. Reduced body mass decreased baseline oxygen demand and improved blood-flow distribution to active muscle groups. Concurrently, exercise enhanced mitochondrial function and muscular oxidative capacity, improving oxygen extraction. Endothelial improvements (via nitric oxide–mediated vasodilation) and reduced vascular resistance further optimized systemic and coronary perfusion under stress. Decreases in arterial pressure, insulin resistance, and metabolic load likely reduced functional coronary stenosis, enabling improved oxygen delivery. Accordingly, aerobic and resistance training has been shown to lower coronary artery disease risk by approximately 30%–40% in primary prevention settings, while participation in structured exercise-based cardiac rehabilitation programs reduces all-cause and cardiovascular mortality by 20%–25% in secondary prevention (Level Ia, Grade A) (34).

To minimize potential adverse effects associated with this intensive program, several precautionary measures must be implemented. Long-term extreme caloric deficits should be avoided, and adequate micronutrient intake (including electrolytes, vitamins, and minerals) must be ensured. Exercise intensity should be progressively increased rather than initiated at moderate to high intensity, with close attention to the patient's physiological compensation. Periodic assessment for refeeding or metabolic rebound phenomena is essential, and weight reduction should follow a gradual, controlled trajectory (≤0.5–1 kg/week) to reduce metabolic adaptation and adverse outcomes (Level Ia, Grade A) (35).

Routine monitoring of electrolytes, renal function, ECG parameters, and cardiac biomarkers (e.g., troponin and NT-proBNP when indicated) is recommended. Adequate protein consumption (≥1.2 g/kg ideal body weight/day) should be maintained to minimize loss of lean body mass (Level III, Grade B) (36). Furthermore, careful evaluation of potential interactions between medications and supplements is required, particularly in patients with cardiovascular disease (e.g., statins and antiplatelet agents). Consideration should also be given to the risk of micronutrient deficiency and the possibility of acute cardiometabolic disturbances (1, 4, 6, 37).

Following completion of the intensive intervention (initial and transition phases), the patient entered a maintenance phase. This phase included sustaining a structured exercise regimen with moderate-intensity aerobic and resistance training while maintaining a high overall training volume. Dietary adjustments emphasized either mild caloric deficit or energy balance to support weight maintenance.

A combined aerobic–resistance program was continued to optimize cardiometabolic benefits, and strategies to minimize metabolic adaptation such as periodic diet breaks, training load variation, and enhancement of non-exercise activity thermogenesis (NEAT) were incorporated. Longitudinal monitoring of metabolic markers (lipid profile, glucose, and relevant hormones) was performed to guide individualized adjustment of dietary and exercise interventions in the event of weight regain or unfavorable metabolic shifts. Importantly, energy restriction during this phase was maintained at a mild, physiologically sustainable level rather than reverting to extreme caloric deficits, alongside continued cardiovascular assessment to ensure safety and clinical stability (20, 38).

Based on the considerations and analyses, the patient completed a ten months intensive lifestyle program, achieving a total weight loss of 50 kg (41% from baseline). This was accompanied by marked improvements in lipid and glycemic parameters, including a substantial increase in HDL-cholesterol (from 0.97 mmol/L to 1.63 mmol/L) and a reduction in HbA1c (from 5.3% to 4.9%). Functional capacity also improved meaningfully, as demonstrated by enhanced exercise stress testing performance, including improved heart rate and blood-pressure responses and a notable increase in MET capacity (from 6.30 to 11.50), reflecting substantial cardiovascular and cardiorespiratory adaptation.

Despite these favorable metabolic and functional changes, a rise in LDL-cholesterol (from 1.51 mmol/L to 3.44 mmol/L) and total cholesterol (from 3.32 mmol/L to 5.47 mmol/L) was observed. This paradoxical lipid elevation likely represents compensatory lipid-metabolism regulation related to rapid weight reduction and mobilization of adipose tissue stores, a phenomenon previously reported in intensive weight-loss interventions (9, 17). Nonetheless, given the patient's coronary risk profile, these changes warrant careful longitudinal evaluation and, if necessary, adjunctive lipid-lowering therapy to mitigate residual atherosclerotic risk (1, 5, 20).

The safety of structured exercise training in patients with coronary artery disease is highly dependent on rigorous patient selection, individualized intensity prescription, and appropriate monitoring. Contemporary evidence emphasizes that hemodynamic surveillance, symptom-guided workload adjustment, and attention to pharmacologic interactions can reduce adverse events during intensive exercise in cardiometabolic disease. As well as TGF-*β*1-induced cardiac fibroblast proliferation, differentiation, and collagen overproduction by modulating the PTEN/Akt/mTOR signaling pathway during exercise. PTEN/Akt/mTOR modulation in cardiometabolic disease is central to its antifibrotic activity (39). Consistent with these recommendations, our program incorporated ECG telemetry during early sessions, predefined blood pressure and ischemia-related termination thresholds, and intensity targets based on percentage heart rate reserve and Borg RPE. The present case therefore supports the concept that appropriately supervised and carefully titrated intensive exercise can be implemented safely even in anatomically severe CAD**,** although broader generalization requires caution.

Recent scientific bulletins stress that lifestyle-centered therapy should not be considered merely adjunctive, but rather an integral therapeutic strategy across the continuum of cardiometabolic disease, including patients with angiographically significant CAD. The present case aligns with these updated perspectives, illustrating that high-adherence, multidisciplinary lifestyle intervention may yield marked functional improvement even in advanced anatomical disease, although decisions regarding revascularization should remain individualized. Accumulating evidence highlights the role of immunometabolic pathways linking obesity, inflammation, and atherosclerosis and fibrosis progression (40). Intensive exercise and weight reduction likely act in part through modulation of these immune-metabolic networks. Although we did not measure cytokines or immune markers, the observed clinical improvements are compatible with favorable alterations in systemic inflammatory activity suggested in prior immunologic research (39, 40).

## Conclusion

A high-intensity exercise approach combined with extreme dietary modification may result in substantial weight reduction and significant improvement in functional capacity. However, current evidence does not yet support the long-term safety and efficacy of such an approach in patients with coronary artery disease (CAD).

Gradual, moderate, and structured interventions aligned with established international guidelines (AHA, ESC, ACC) remain the preferred strategy, supported by robust evidence (Level Ia, Grade A recommendations). Intensive programs of this nature should only be considered in carefully selected patients with adequate clinical stability, under comprehensive multidisciplinary supervision and rigorous monitoring of clinical status and biomarkers.

Furthermore, appropriate pharmacotherapy in accordance with guideline recommendations and evidence-based supplementation are essential to ensure safety and optimize therapeutic benefit. Ongoing evaluation is required to notice the immunometabolic respon on cardiovascular, and pharmacology and nutritional side effects during the intervention process.

## Learning point

Extreme lifestyle therapy is not routine in CAD but may be possible in carefully selected patients.

Close cardiovascular monitoring is essential to avoid ischemia and arrhythmia.

Rapid weight loss may temporarily raise LDL due to fat mobilization.

Guideline-based moderate lifestyle therapy remains first-line; extreme programs belong only in supervised settings.

## Limitations

This is a single patient observation without control group. Therefore causality cannot be inferred. The protocol applied in this case is not guideline standard and should not be interpreted as evidence for routine use in CAD patients. Hormonal and advanced lipid testing (apo-B, lipoprotein subfractions) were not performed. In this study, cardiopulmonary exercise testing was not available, therefore VO₂ peak and ventilatory efficiency variables could not be obtained and physiologic adaptations were inferred from exercise treadmill test performance. Physiologic adaptation was inferred from ETT parameters. Imaging follow-up of coronary anatomy was not repeated, so plaque regression or progression cannot be determined. Diet composition, statin adherence variability, and genetic lipid responsiveness confound the interpretation of LDL changes. Therefore, generalizability is limited and findings should be interpreted cautiously.

## Patient perspective

When I first learned about the severity of my coronary artery condition, I felt anxious but determined to avoid surgery if possible. I declined the PCI procedure because the risk of lifelong anticoagulant use after PCI and the surgery cost. I wanted to prove to myself that I could change my lifestyle and take control of my health. I decided to pursue a non-procedural approach and committed fully to the intensive program recommended by my clinical team.

The early phase was very challenging. Adjusting to strict nutrition changes and high-intensity exercise was physically exhausting and mentally demanding. There were moments when I felt weak and doubted whether I could continue. However, the gradual improvements in my breathing, stamina, blood pressure, and overall energy motivated me to stay consistent.

Progress did not happen overnight. It required discipline, patience, and continuous monitoring by my doctors. As my body changed, I felt lighter, more confident, and capable of activities I had not imagined doing before. This journey taught me that extreme programs require careful supervision and personal commitment. The most important lesson for me is that meaningful improvement is possible with the right guidance, consistent discipline, and strong support from healthcare professionals.

Now, I feel proud of my progress and more aware of maintaining my health long-term. I am grateful for the multidisciplinary team that guided me safely through this journey.

## Data Availability

The original contributions presented in the study are included in the article/Supplementary Material, further inquiries can be directed to the corresponding author/s.
